# A dual-functional lanthanide nanoprobe for both living cell imaging and ICP-MS quantification of active protease†
†Electronic supplementary information (ESI) available: Experimental procedures, characterization data, and supporting figures. See DOI: 10.1039/c5sc03363b


**DOI:** 10.1039/c5sc03363b

**Published:** 2015-12-15

**Authors:** Duan Feng, Fang Tian, Weijie Qin, Xiaohong Qian

**Affiliations:** a National Center for Protein Sciences Beijing , State Key Laboratory of Proteomics , Beijing Proteome Research Center , Beijing Institute of Radiation Medicine , Beijing , China 102206 . Email: aunp_dna@126.com ; Email: qianxh1@163.com

## Abstract

Real-time imaging and quantitative monitoring of dynamic changes in biomolecules in bioprocesses is in urgent need for biological and clinical studies. However, this goal is difficult to achieve due to the incompatibility between the commonly used imaging and quantification methods. To fulfill this need, we developed the first nanoprobe capable of both living cell imaging and ultrasensitive quantification of proteases in cancer cells by taking advantage of the characteristic luminescence and ICP-MS response of lanthanide metals. The nanoprobe is composed of a lanthanide metal-based luminescent donor/mass tag and a gold nanoparticle quencher linked by a specific peptide recognized by proteases. The protease activity in living cells is both visualized and quantified by monitoring the enzymatically released lanthanide metal using fluorescence imaging and ICP-MS. The low ppb level sensitivity of this method demonstrates its potential in the study of protease-dependent pathways and related diseases.

## Abstract 2

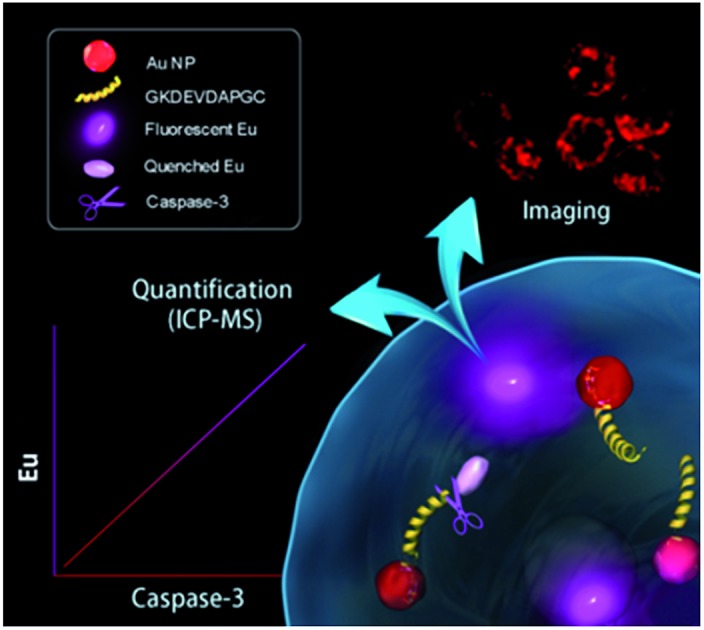
A novel nanoprobe is successfully developed for simultaneous living cell imaging and sensitive quantification of caspase-3 in cancer cells.

## Introduction

Fluorescence imaging is currently one of the most widely adopted methods to monitor biomolecules and processes in real-time^1^ and provides direct visualization of biological analytes in their native state within living biological systems.^2^ Though offering high sensitivity and unrivaled spatiotemporal resolution, fluorescence imaging still faces difficulties in quantitative detection. First, the emission properties of fluorescent probes may be sensitive to environmental changes,^3^ which makes accurate quantitative measurement difficult. Second, the auto-fluorescence of biological systems may also interfere with the quantitative detection of biomolecules.^4^ To accurately determine the functional variations of many key biomolecules, it is desirable to both visualize their subcellular location and quantify their concentration and activity changes under different conditions.^5^ Thus, developing a method capable of both sensitive real-time imaging and accurate quantification for biomolecule detection is highly demanded and remains challenging.

As one of the methods of choice for accurate quantification, inductively coupled plasma mass spectrometry (ICP-MS) has increased in popularity in biological research in recent years, due to its ppt level sensitivity and nine orders of magnitude dynamic range.^6^ Successful applications of ICP-MS have been reported for cell, protease and DNA analysis *via* elemental tagging,^7^ demonstrating its unique advantages as an ideal quantification method, though no real-time information is provided. Therefore, a new method that combines fluorescence imaging and ICP-MS quantification would integrate the advantages of the two analytical techniques and promote our understanding on the functional variation of biomolecules in biological processes.

Here, we report the design and synthesis of the first lanthanide metal-based, dual-functional nanoprobe for living cell imaging and sensitive quantification of protease activity by exploiting both the characteristic luminescence and excellent ICP-MS response of lanthanide metals. Fluorescence imaging with low background interference is achieved using a fluorescence resonance energy transfer (FRET) system comprising a lanthanide metal-based luminescent donor and a gold nanoparticle (Au NP) quencher linked by a specific peptide recognized by the selected proteases. Activation of the proteases results in the proteolysis of the peptide linker and can be monitored *via* the restoration of the luminescence of the released lanthanide metal. More importantly, the activity of the proteases under different conditions can also be accurately and sensitively determined by quantifying the released lanthanide metal using ICP-MS.

Caspases are proteases that catalyze the proteolysis of peptide bonds and initiate specific degradation of many key cellular proteins, which lead to cell apoptosis. Among caspases, caspase-3 is the central mediator in the initiation and propagation of cell apoptosis, and higher levels of activated caspase-3 in tumors are correlated with an increased rate of recurrence and death.^8^ Herein, a large number of fluorescence based methods have been reported for caspase-3 analysis.^9^ Some of them offered fluorescence imaging of endogenous caspase-3,9a,9b while others provided absolute quantification of exogenous caspases-3 with a 20 pM detection limit,9*c* but none of them achieved both. To better understand the key roles of caspase-3 in cell apoptosis and cancer development, it is crucial to develop new probes that can not only sensitively detect endogenous caspase-3 in real-time, but also accurately quantify the dynamic changes in caspase-3 activity at the same time. Such information could directly and accurately determine the functional variations of caspase-3 and other proteases and provide tools and evaluation standards for the diagnosis of disease, monitoring of the therapeutic effect of cancer treatment or efficacy of new drugs.

## Results and discussion

### Design of the nanoprobe

**Scheme 1:**
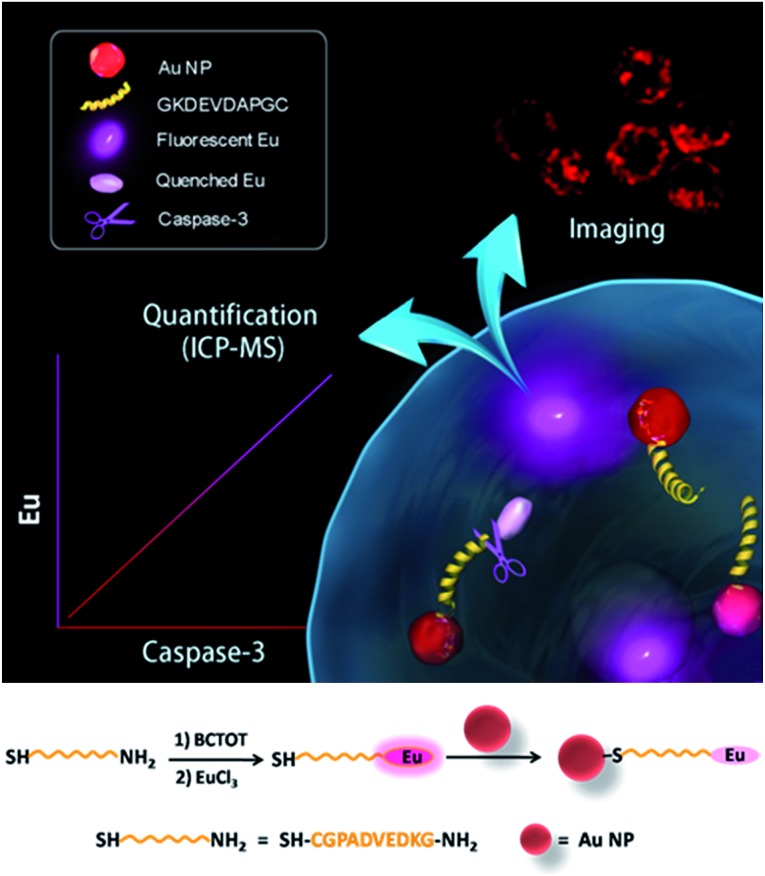
Application of the lanthanide metal-based dual-functional nanoprobe for living cell imaging and ICP-MS quantification of caspase-3 (upper). Synthesis of the lanthanide metal-based nanoprobe (bottom).

The design of the dual-functional probe is shown in Scheme 1. The nanoprobe is composed of europium (Eu) metal chelated by BCTOT (1,10-bis(5′-chlorosulfo-thiophene-2′-yl)-4,4,5,5,6,6,7,7-octafluorodecane-1,3,8,10-tetraone), a caspase-3-specific peptide substrate linker and an Au NP quencher. The “smart” design of this probe is the choice of Eu metal as the signal response moiety, which provides both an “off–on” luminescence signal *via* FRET and a mass tag in ICP-MS analysis. Eu metal, which possesses a large Stokes shift and line-like emission spectra, has long been favored for the development of luminescent probes.^10^ Eu is also a particularly attractive option as a mass tag for absolute protein quantification due to its sensitive response in ICP-MS and low background interference in biological samples.^11^ Furthermore, in this nanoprobe, the Au NP is not only an efficient quencher for Eu luminescence but also serves as a “carrier matrix” that enables facile separation and removal of the uncleaved Eu chelated peptides by centrifugation. Therefore, this strategy for living cell monitoring and accurate quantification of caspase-3 activity could be expanded to the analysis of other key enzymes, such as nucleic acids or protein-modifying enzymes, by designing a chelated Eu probe with the recognition sequence of the corresponding enzyme.

To design the dual-functional nanoprobe (Scheme 1), a specific peptide (GKDEVDAPGC) substrate containing the core recognition sequence (DEVD) of caspase-3 (ref. 12) was first selected. Second, the antenna chromophore BCTOT was synthesized to chelate Eu and label the peptide substrate *via* the reaction between the SO_2_Cl of BCTOT and the NH_2_ of the peptide.13*a* Finally, the nanoprobe was prepared by introducing the Eu–BCTOT tagged peptide onto the surface of the Au NP through the formation of a stable Au–S covalent bond between the C-terminal SH of the peptide and the Au NP.^14^ In the nanoprobe, the luminescence of Eu–BCTOT is “off” due to the energy transfer to the Au NP quencher. However, when the nanoprobe enters the cell, the Eu–BCTOT-labeled peptide is specifically cleaved by endogenous caspase-3, resulting in the release of the Eu–BCTOT–peptide fragment from the Au NP and turning on the luminescence. After cell lysis and centrifugation, the cleaved Eu–BCTOT–peptide fragment in the supernatant is separated from the uncleaved Eu–BCTOT–peptide, which remains attached to the Au NP precipitated in the bottom of the test tube; the cleaved Eu–BCTOT–peptide fragment in the supernatant is subjected to ICP-MS analysis. In this way, the activity of caspase-3 under specific cellular states can be monitored in real-time by the luminescence recovery of Eu–BCTOT and can be accurately quantified using the Eu signal in ICP-MS.

### Synthesis and spectroscopic properties of the nanoprobe

To construct the nanoprobe, BCTOT was synthesized following the reported procedure^13^ and characterized using ^1^H NMR spectroscopy (Fig. S1†). The maximum excitation wavelength of Eu–BCTOT is 350 nm (Fig. S2a†). The fluorescence spectra reveal that the mixture of Eu and BCTOT exhibits a sharp emission at 614 nm upon excitation at 350 nm (Fig. S2b†), which is the characteristic emission of Eu–BCTOT and demonstrates the successful chelation of Eu with BCTOT and the luminescence properties of Eu–BCTOT. Next, the conditions for preparing Eu–BCTOT were optimized to achieve the highest emission efficiency. The fluorescence of Eu–BCTOT gradually increases as the ratio between Eu and BCTOT increases from 0.1 : 1 to 1 : 1 and reaches a plateau at 1 : 1 (Fig. S3†). The strongest fluorescence is obtained at 10 μM Eu–BCTOT (Fig. S4†). In addition, the fluorescence intensity of Eu–BCTOT at 614 nm increases with chelation time and reaches a plateau after 1 h (Fig. S5†). No obvious fluorescence change is observed after 24 h, indicating that the Eu–BCTOT complex is stable.

After obtaining the optimized fluorescence conditions, the Eu–BCTOT complex was covalently conjugated with the peptide substrate of caspase-3, and the Eu–BCTOT–peptide was conjugated to the Au NP to prepare the final FRET pair. As revealed by the transmission electron microscopy images, the average particle size of the Au NP increases from 13.6 nm to 16.2 nm after conjugation with the Eu–BCTOT–peptide, with no apparent particle aggregation (Fig. S6†). After conjugation, the initial strong fluorescence is nearly completely quenched by the 13 nm Au NP as a result of efficient energy transfer from the Eu–BCTOT to the Au NP (Fig. S7†), demonstrating that Eu–BCTOT–peptide was successfully conjugated to the Au NP.

### Fluorescence response of the nanoprobe to caspase-3

**Fig. 1:**
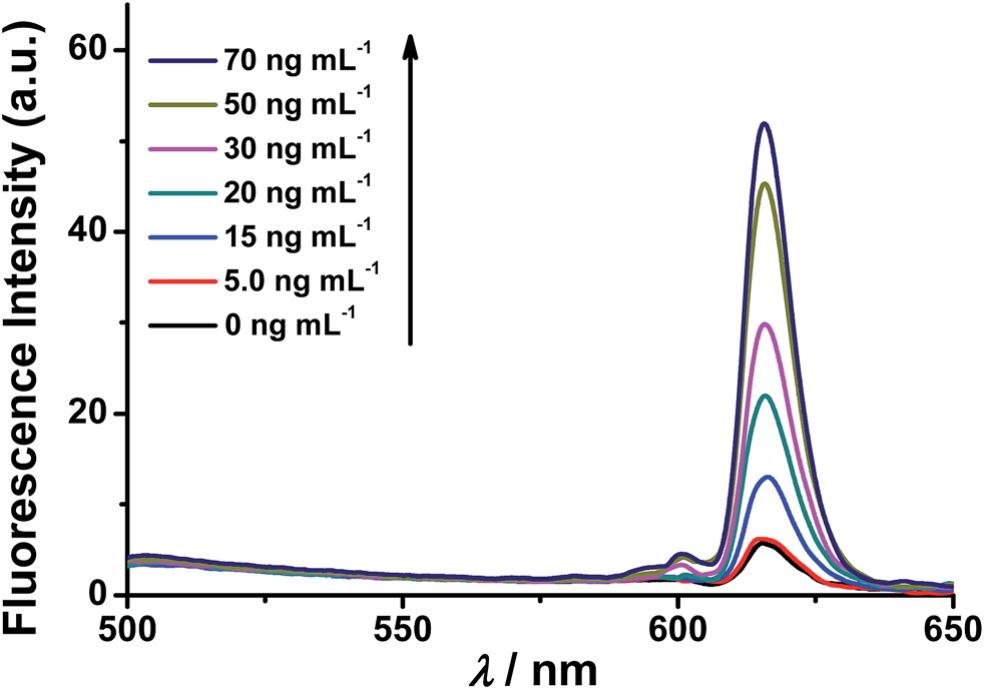
Fluorescence spectra of the nanoprobe in the presence of different concentrations of caspase-3 (0.0, 5.0, 15, 20, 30, 50 and 70 ng mL^–1^). The reaction of the nanoprobe with caspase-3 was performed at 37 °C for 2 h in PBS buffer, then the resulting solution was centrifuged, and the supernatant was subjected to fluorescence measurements. *λ*_ex_ = 350 nm.

The fluorescence response of the synthesized nanoprobe to caspase-3 was then investigated. As shown in Fig. 1, as expected, the fluorescence intensity of the nanoprobe increases with increasing caspase-3 concentration from 5 to 70 ng mL^–1^, demonstrating that efficient FRET-based off–on fluorescence is achieved for caspase-3 sensing. To confirm that the fluorescence enhancement originates from the cleavage of the peptide substrate DEVD by caspase-3, Ac-DEVD-CHO, a caspase-3 inhibitor, was introduced to alter the activity of caspase-3. In the presence of Ac-DEVD-CHO, only a very weak fluorescence intensity is observed after incubating the nanoprobe with caspase-3 (Fig. S8†), suggesting that the fluorescence recovery originates from the caspase-3 cleavage of the peptide substrate of the nanoprobe and is directly correlated with the actual activity of caspase-3. To further evaluate the cleavage selectivity of caspase-3 toward the nanoprobe, a control nanoprobe with the peptide substrate sequence DEVG was used. The single amino acid change from D to G in the peptide substrate leads to nearly complete suppression of the caspase-3 cleavage, and only negligible fluorescence recovery from the nanoprobe FRET pair is observed (Fig. S8†). Therefore, the recognition specificity of the nanoprobe for caspase-3 is valid and strongly supports selective caspase-3 detection.

### ICP-MS response of the nanoprobe to caspase-3

**Fig. 2:**
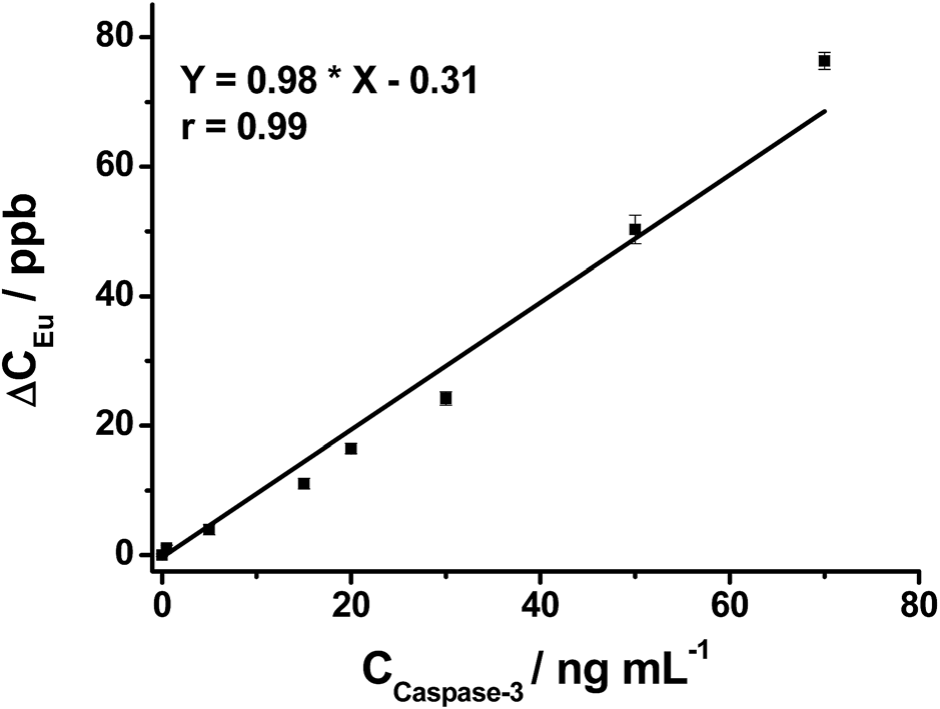
The linear relationship between Δ*C*_Eu_ and concentration of caspase-3 (0.0, 0.5, 5.0, 15, 20, 30, 50 and 70 ng mL^–1^). Δ*C*_Eu_ is the difference between the Eu concentrations in the supernatant (acquired by ICP-MS) before and after caspase-3 treatment. Data are expressed as the mean of three separate measurements ± SD.

After demonstrating the successful off–on fluorescence of the nanoprobe *via* selective peptide cleavage by caspase-3, the released Eu was further analyzed to obtain quantitative information about caspase-3 activity by ICP-MS. The Eu response in ICP-MS for varying concentrations of caspase-3-treated nanoprobe displays a trend similar to that of fluorescence. As shown in Fig. 2, the ICP-MS signal of the cleaved peptide tagged with Eu increases with the increasing caspase-3 concentration, and a strong linear correlation of [Eu] (ppb) = 0.98 × [caspase-3] (ng mL^–1^) – 0.31 (*r* = 0.99) is obtained within a two order of magnitude range from 0.5 to 70 ng mL^–1^, demonstrating the capability of the nanoprobe for quantitative detection of caspase-3.

Potential interfering substances in living systems may interfere with the stability of the nanoprobe and induce false positive results. Therefore, the Eu concentration in the supernatant of the nanoprobe treated with various potential interfering substances was examined by ICP-MS. The nanoprobe produces the highest Eu concentration in the presence of caspase-3. In contrast, other substances including two enzymes, trypsin and caspase-8 generate at least a three times lower Eu concentration (Fig. S9†), which is quite close to that of the blank sample (nanoprobe in PBS), indicating the non-caspase-3 released Eu is negligible.

### Fluorescence imaging of endogenous caspase-3 in living cells

**Fig. 3:**
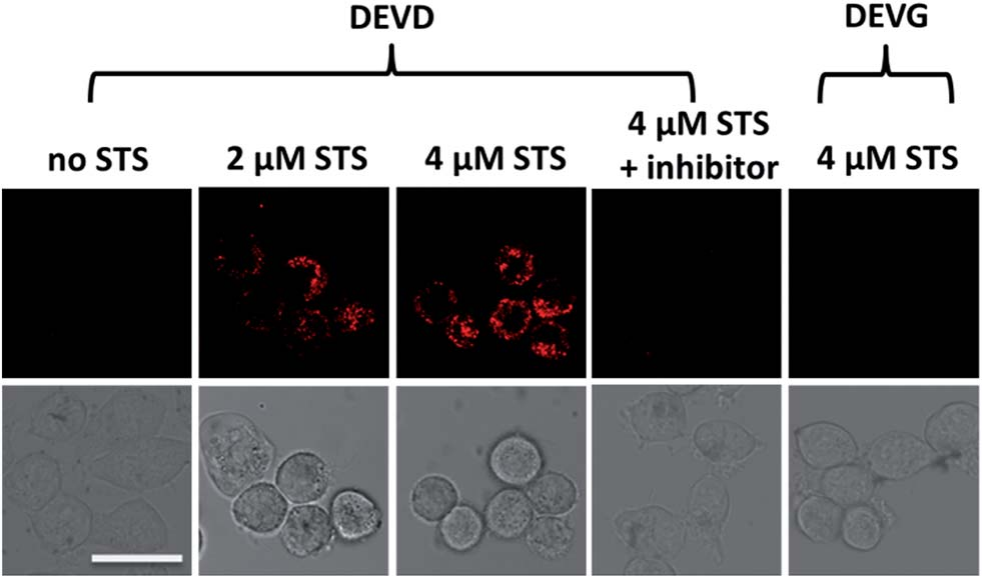
Confocal fluorescence images of HeLa cells under different treatments. HeLa cells were incubated with caspase-3 specific peptide DEVD modified nanoprobe or nonspecific peptide DEVG modified control-nanoprobe, and varied concentrations of STS or Ac-DEVD-CHO. The differential interference contrast (DIC) images of the corresponding samples are shown at the bottom. Scale bar = 25 μm.

After successfully demonstrating the high selectivity, stability and quantification capability of the nanoprobe for exogenous caspase-3 analysis, we further applied it to the challenge of living cell imaging and concentration/activity determination of endogenous caspase-3. HeLa cells were treated with the nanoprobe (DEVD) or the control-nanoprobe (DEVG) with varied concentrations of staurosporine (STS, an apoptosis inducer) or Ac-DEVD-CHO (inhibitor of caspase-3). Confocal fluorescence microscopy images of HeLa cells are presented in Fig. 3. Without treatment, the HeLa cells display only negligible fluorescence after incubation with the nanoprobe, indicating very limited caspase-3 concentration/activity in the normal cell state. However, in the presence of 2 μM STS, fluorescence from the cleaved Eu–BCTOT–peptide fragment is observed in the cells and even stronger fluorescence is obtained at a higher concentration (4 μM) of STS. This increase in fluorescence can be attributed to the further activation of caspase-3 by STS.^15^ Furthermore, the observed fluorescence is primarily localized in the cytoplasm, indicating the subcellular location of the caspase-3 induced by STS, consistent with the location reported in the literature.^16^ By contrast, HeLa cells treated with 4 μM STS and Ac-DEVD-CHO generate insignificant fluorescence, which can be explained by the efficient inhibition of caspase-3 by Ac-DEVD-CHO. Meanwhile, HeLa cells incubated with the control-nanoprobe (DEVG) generate no obvious fluorescence, even in the presence of 4 μM STS, which confirms that the fluorescence signal results from the specific cleavage of DEVD by caspase-3. The above results demonstrate that the FRET-based “off–on” fluorescence and high selectivity of the nanoprobe are valid in living cells for endogenous caspase-3 analysis.

### Quantification of endogenous caspase-3 by ICP-MS

To accurately quantify the actual level of active endogenous caspase-3, the amount of the Eu–BCTOT–peptide fragment released from the nanoprobe by caspase-3 cleavage was quantified by ICP-MS after cell lysis. After centrifugation, the Eu signal in the supernatant was measured by ICP-MS and was converted to the concentration of active caspase-3 using the linear equation obtained using exogenous caspase-3 (Fig. 2). The data are presented in Table 1. Under normal conditions, the concentration of active caspase-3 in HeLa cells determined by the nanoprobe is 1.0 ng mL^–1^ at a density of 2.00 × 10^6^ cells per mL, indicating the high sensitivity of this quantification method, which reaches low ppb levels. A proportional increase in the concentrations of active caspase-3 is observed when HeLa cells were treated with increasing concentrations of STS, in agreement with the fluorescence imaging observations. Simultaneous introduction of STS and Ac-DEVD-CHO leads to a reduced Eu signal, which suggests the activity of caspase-3 is inhibited. Similarly, the Eu signal obtained from the control-nanoprobe and STS-treated cells is lower due to the substrate specificity of caspase-3, which is unable to cleave DEVG. These findings further demonstrate that the uncleaved Eu–BCTOT–peptides attached on Au NP can be efficiently separated from the cleaved peptide fragments by centrifugation and do not interfere with the quantification of active caspase-3. The above study provides the first absolute quantification of active caspase-3 in HeLa cells and may facilitate the functional study of caspase-3 and the related dysregulation of apoptosis in a variety of human diseases. Furthermore, the manipulation of caspase-3 by STS and Ac-DEVD-CHO can be accurately and sensitively monitored *via* the corresponding change in Eu, providing a facile method for activity enhancer or inhibitor screening for the caspase family, which is a known therapeutic drug target for cancer, autoimmune disorders and heart disease.^17^

**Table 1:** Quantification of active caspase-3 in HeLa cells under different treatments

Treatment	Concentration of caspase-3 found (ng mL^–1^; *n* = 3)	Standard deviation
HeLa cells alone	<0.5 (below linear range)	—
DEVD	1.0	0.0330
DEVD + 2 μM STS	1.3	0.0451
DEVD + 4 μM STS	1.8	0.0298
DEVD + 4 μM STS + inhibitor	<0.5 (below linear range)	—
DEVG + 4 μM STS	<0.5 (below linear range)	—

## Conclusions

In summary, we successfully developed a simple and robust nanoprobe labeled with Eu for both living cell imaging and highly sensitive quantification of active caspase-3 in cancer cells. This is the first study to demonstrate the feasibility of a lanthanide metal-based nanoprobe for the simultaneous visualization and quantification of protease in real biosystems and to determine the absolute quantity of active caspase-3 in cell apoptosis. We expect this simple design for a nanoprobe to be applicable as a general strategy for monitoring the expression and activity of other enzymes.

## Supplementary Material

Supplementary information
