# Supplementary material for: A dual-functional lanthanide nanoprobe for both living cell imaging and ICP-MS quantification of active protease
Source: Chem Sci. 2015 Dec 15;7(3):2246–50. doi: 10.1039/c5sc03363b (PMC5977404; doi:10.1039/c5sc03363b)
Supplement: Supplementary file 1 [file SC-007-C5SC03363B-s001.pdf]

Electronic Supplementary Material (ESI) for Chemical Science.
This journal is © The Royal Society of Chemistry 2016

Electronic Supplementary Information for

A dual-functional lanthanide nanoprobe for both living cell imaging and ICP-MS quantification of active protease†

Duan Feng, Fang Tian, Weijie Qin* and Xiaohong Qian*

National Center for Protein Sciences Beijing, State Key Laboratory of Proteomics, Beijing Proteome Research Center, Beijing Institute of Radiation Medicine, Beijing 102206 (China)

Materials and apparatus

Europium (III) chloride ($EuCl_3$), caspase-3, caspase-8, trypsin, Ac-DEVD-CHO, staurosporine (STS), bovine serum albumin (BSA) and human serum albumin (HSA) were obtained from Sigma-Aldrich (St. Louis, MO, USA). The caspase-3 specific peptide with a sequence of GKDEVDAPGC, and the control peptide with a sequence of GKDEVGAPGC were purchased from Beijing SBS Genetech Co., Ltd (Beijing, China). $HAuCl_4$, trisodium citrate, tetrahydrofuran (THF), NaCl, KCl, $CaCl_2$, $MgCl_2$, glucose, glutamine, serine, arginine, vitamin B1 and glutathione (GSH) were purchased from Beijing Chemicals, Ltd (Beijing, China). Other reagents employed were all of analytical grade, and were used without further purification. A phosphate buffered saline (PBS; 137 mM NaCl, 2.7 mM KCl, 4.3 mM Na_2HPO_4, 1.4 mM KH_2PO_4, pH 7.4) solution was employed in the experiments. Deionized and distilled water was used throughout.

A Hitachi F-2500 spectrofluorimeter (Hitachi, Japan) was used for fluorescence measurements. UV-vis spectra were recorded using a 1-cm quartz cells and a TU-1900 spectrophotometer (Beijing Purkinje General Instrument Co., Ltd, Beijing, China). The sizes of Au NPs were determined by transmission electron microscopy (TEM, JEM-1011, JEOL, Japan). Fluorescence imaging was performed using a Leica TCS SP5 inverted confocal microscope (Leica, Germany). The cellular images were acquired using a 40 × objective. Ar^+ laser (405 nm) was used as excitation source for Eu-BCTOT complex, and a 550-650 nm bandpass filter was used for fluorescence detection.

The quantitation of europium was carried out on an Agilent 7700x series inductively coupled plasma mass spectrometer (USA) with the following conditions: RF plasma source: 1550 W; Ar plasma gas: 15 L min^{-1}; Ar carrier gas: 1.07 L min^{-1}. The samples were introduced via a Micromist nebulizer. An octopole reaction/collision cell (ORS^3 system) was involved to remove the polyatomic interferences (He collision gas 4.0 mL min^{-1}). The ions were then separated by a quadrupole mass analyser and measured by a Discrete Dynode Electron Multiplier (DDEM) detector. The isotope monitored for Eu is ^{153}Eu, which is not interfered by any other atoms. The sample

solutions were stored at 277 K before ICP-MS measurement and tested as soon as possible. A mixed standard solution of rare earth metals (10 ppm) was obtained from Agilent (USA). Different concentrations (0.01, 0.1, 1, 10, 100 ppb) of standard solution were newly prepared by diluting the original standard solution with 1% HNO_3 (trace metal level, obtained as 68-70% HNO_3 from Thermo Fisher, UK, diluted with purified water from a Millipore water purifying system, US). The data were the average of three parallel measurements.

Synthesis of BCTOT

BCTOT (1,10-bis(5'-chlorosulfo-thiophene-2'-yl)-4,4,5,5,6,6,7,7-octafluorodecane-1,3,8,10-tetraone) was prepared following the reported procedure.[S1, S2] In brief, 4 g of $NaOCH_3$ was added over 10 min in several batches to a flask containing 5 g of dimethyl octafluoroadipate and 4 g of 2-acetylthiophene in 50 mL of dry ether. After stirring at room temperature for 48 h, the mixture was poured into 200 mL of 15% H_2SO_4 solution. The ether phase was separated and distilled at reduced pressure. The solid was recrystallized in anhydrous ethanol, filtered off, and vacuum dried in the presence of P_2O_5 to obtain BTOT (1,10-bis(thiophene-2'-y l)-4,4,5,5,6,6,7,7-octafluor odecane-1,3,8,10-tetraone). Under stirring, 1.6 g of BTOT was added to a flask containing 10 mL of $ClSO_3H$. After stirring the mixture at 40 °C for 3 h, the solution was added dropwise to ice-cold water, and the product BCTOT was extracted with ether. Next, the ether was distilled to ~0.5 mL and the precipitate was filtered off and recrystallized in anhydrous ether. Finally, a grey powder of BCTOT was obtained. The product was stored under argon in small glass bottles in a desiccator at -20 °C. BCTOT was characterized by ^1H NMR. ^1H NMR (400 MHz, $CDCl_3$) δ 7.89 (d, J = 4.2 Hz, 2H), 7.78 (d, J = 4.2 Hz, 1H), 6.54 (s, 2H).

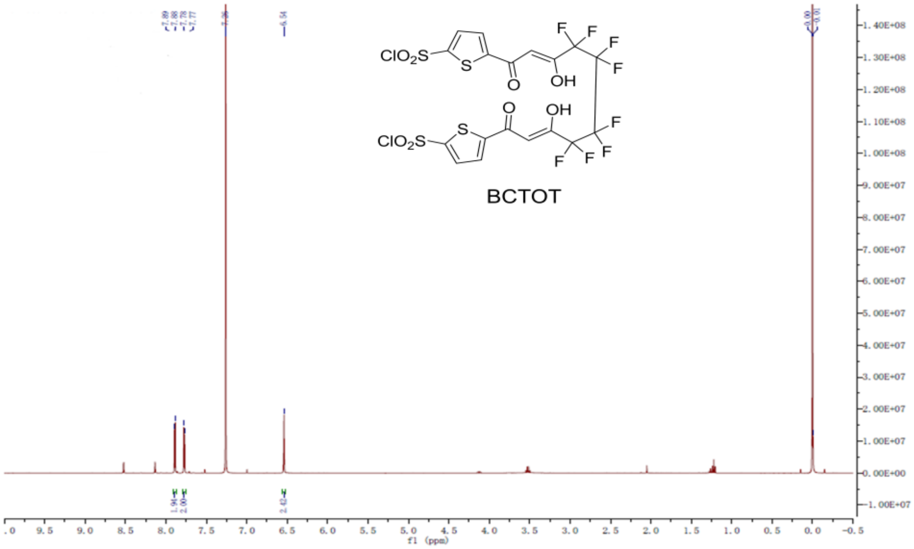

Fig. S1 The molecular structure and ^1H NMR spectrum of BCTOT.

Optimization of the conditions for chelation of Eu with BCTOT

$EuCl_3$ was dissolved in deionized water and added into the solution of BCTOT. After incubating at 50 °C for varied periods of time, the spectroscopic properties of the chelated Eu-BCTOT complex were measured. The

maximal excitation and emission wavelengths of the Eu-BCTOT complex were at 350 nm and 614 nm, respectively. Maximum fluorescence intensity was obtained at a Eu/BCTOT ratio of 1.0 at a concentration of 10 μM.

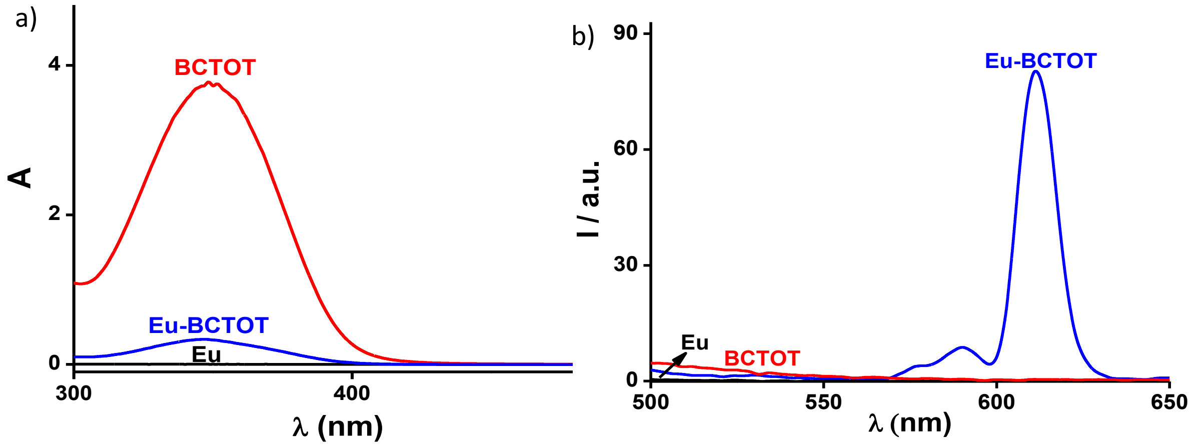

Fig. S2 a) Absorption and b) fluorescence emission spectra of Eu, BCTOT and Eu-BCTOT complex (molar ratio of Eu/BCTOT = 1:1) at a concentration of 10 μM, respectively. λ_{ex} = 350 nm.

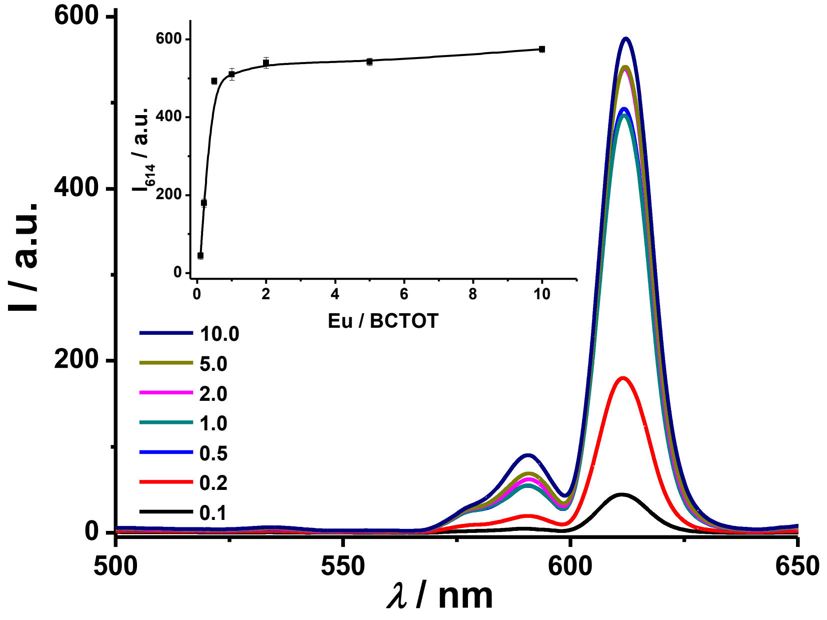

Fig. S3 Fluorescence spectra of Eu-BCTOT complex at different Eu/BCTOT ratios. The inset shows the fluorescence intensity of Eu-BCTOT complex with varied molar ratios of Eu/BCTOT. λ_{em} = 614 nm; data are expressed as the mean of three separate measurements ± standard deviation (SD).

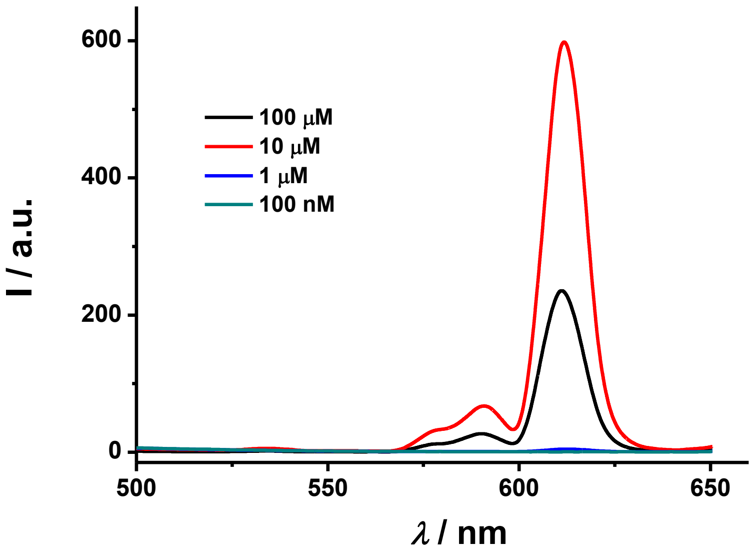

Fig. S4 The fluorescence spectra of different concentrations of Eu-BCTOT complex (molar ratio of Eu/BCTOT = 1:1).

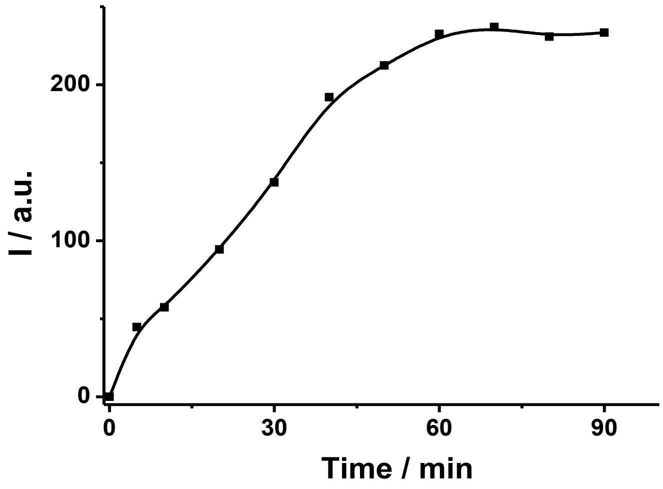

Fig. S5 The fluorescence intensities of Eu-BCTOT complex (molar ratio of Eu/BCTOT = 1:1) at different chelation time in PBS buffer at 50 °C. $\lambda_{ex/em}$ = 350/614 nm.

Preparation of Eu-BCTOT complex modified peptide

The BCTOT modified peptide was prepared by the reaction of BCTOT with the synthesized peptides via the formation of a covalent SO_2-NH bond between the SO_2Cl group of BCTOT and the N-terminal NH_2 of the peptide. Briefly, BCTOT (1.5 μmol) was incubated with the synthesized peptide in a molar ratio of 4:1 (BCTOT/peptide) in carbonate buffer (0.1 M, pH 9.0) at room temperature for 1 h.

Next, $EuCl_3$ was dissolved in deionized water and then added into the BCTOT modified peptide solution, and incubated at 50 °C for 2 h. The maximal excitation and emission wavelengths of the obtained highly fluorescence Eu-BCTOT-peptide were at 350 nm and 614 nm, respectively.

Preparation and characterization of Eu-BCTOT-peptide modified Au NPs

Au NPs of 13 nm were prepared by the trisodium citrate reduction of $HAuCl_4$ following the reported method.[S3] Briefly, 50 mL of aqueous $HAuCl_4$ (0.25 mM) solution was heated to reflux with vigorous stirring and 1.3 mL of trisodium citrate (1%, w/v) was then added into the solution rapidly. The color of solution changes from light yellow to wine red in about 3 min, indicating the formation of Au NPs. The solution was cooled to room temperature with continuous stirring and then stored in a refrigerator at 4°C overnight prior to use. The concentration of the as-prepared Au NPs was determined to be 3 nM by a molar absorptivity of 2.7×10^8 M^{-1} cm^{-1} at 518 nm for 13 nm Au NPs.

Next, the Eu-BCTOT-peptide was immobilized on Au NPs to obtain the nanoprobe. The strong affinity of the SH group of the C-terminal cysteine residue of the Eu-BCTOT-peptide toward gold enables efficient immobilization of the peptide on the surface of Au NPs via the formation of the stable Au-S bond. In brief, the Eu-BCTOT-peptide and Au NPs solution were mixed at a molar ratio of 3000/1 (peptide/Au NPs). After gently stirring for 48 h at room temperature, excess Eu-BCTOT-peptides were removed by repeated centrifugation (12000 rpm × 10 min, three times) and washing with deionized water. The obtained red precipitate was suspended in fresh PBS buffer (pH 7.4).

The sizes of Au NPs were determined by TEM. Samples for TEM analysis were prepared by placing 20 µL of Au NPs solution on the carbon-coated copper grid and then drying at room temperature. The average diameters of the monodisperse unmodified and Eu-BCTOT-peptide modified Au NPs are 13.6 nm and 16.2 nm, respectively.

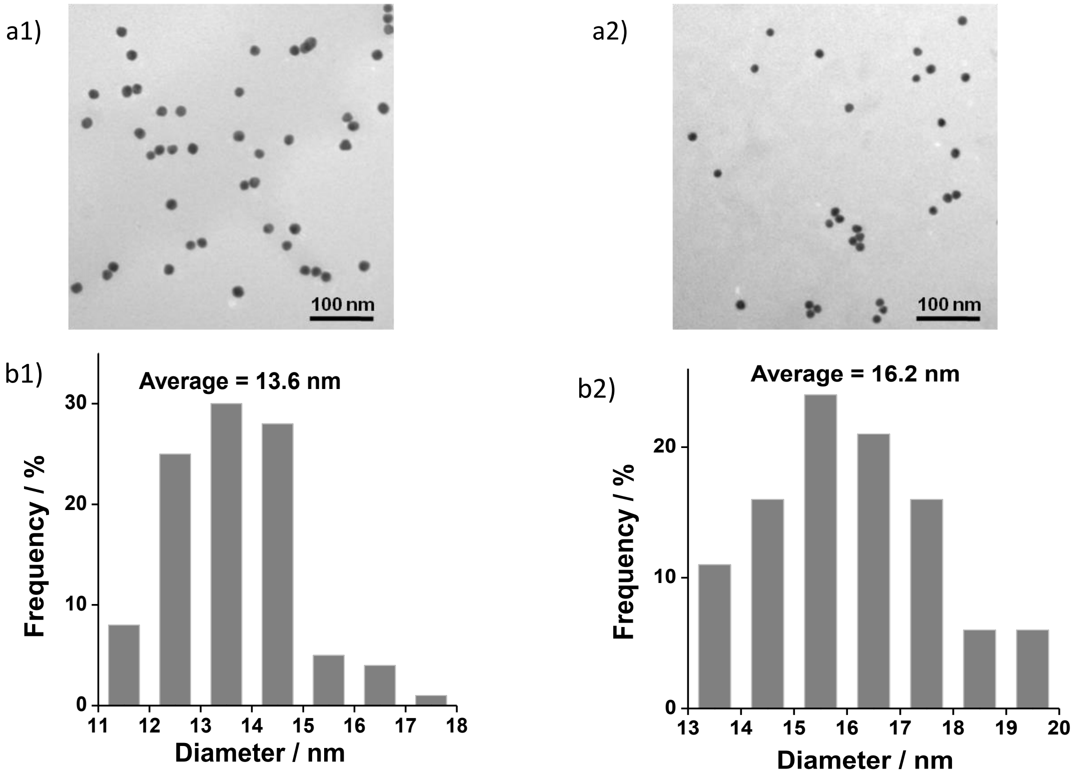

Fig. S6 The TEM images (above) and size distribution histograms (below) of unmodified Au NPs (a1 and b1) and Eu-BCTOT-peptide modified Au NPs (a2 and b2). Each of the histograms was made from the TEM images by counting 100 particles.

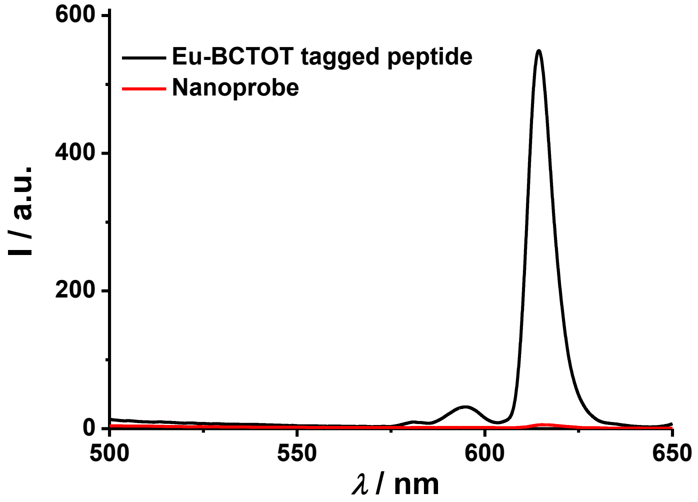

Fig. S7 Fluorescence spectra of the Eu-BCTOT tagged peptide before and after conjugation with Au NPs (nanoprobe) in PBS buffer. λ_{ex} = 350 nm.

Fluorescent detection of exogenous caspase-3

For fluorescence analysis, different concentrations of exogenous caspase-3 were incubated with 10 μM nanoprobe in the PBS buffer at 37 °C for 2 h. After reaction, the resulting solution was centrifuged, and the supernatant was subjected to fluorescence measurements. Fluorescence intensity/spectrum of each sample was then recorded with $\lambda_{ex/em}$ = 350/614 nm.

To confirm that the fluorescence enhancement originates from the cleavage of the DEVD by caspase-3, 10 μM nanoprobe (DEVD) was incubated with 70 ng mL^{-1} caspase-3 in the presence and absence of Ac-DEVD-CHO at 37 °C for 2 h, and then the supernatant was subjected to fluorescence measurements. The fluorescent response of 10 μM control-nanoprobe (DEVG) to caspase-3 was tested under the same conditions. The fluorescence spectrum of each sample was recorded with $\lambda_{ex/em}$ = 350/614 nm and the results were shown in Fig. S8.

For quantitative determination, the same experimental operation was conducted as mentioned above. The supernatant was collected and analyzed by ICP-MS.

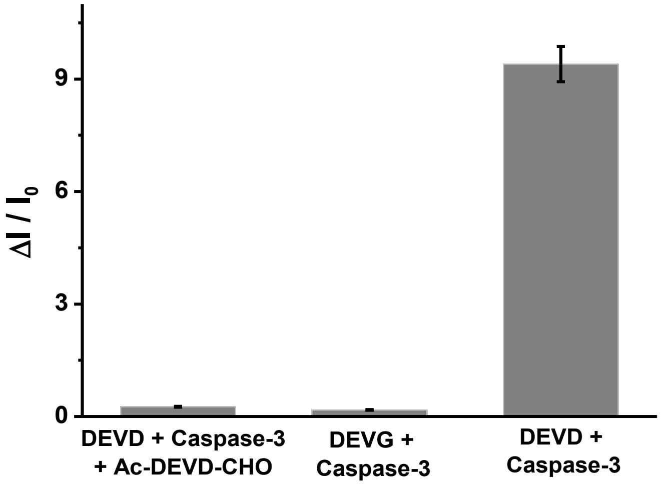

Fig. S8 Fluorescence responses of the nanoprobe under different conditions. Nanoprobe with inhibitor Ac-DEVD-CHO (100 μM) plus caspase-3 (70 ng mL^{-1}), control-nanoprobe with caspase-3, and nanoprobe with caspase-3. I_0 represents the initial fluorescence intensity of the nanoprobe/control nanoprobe solution, and Δ*I* is the changes in the fluorescence intensity after caspase-3 treatment. Data are expressed as the mean of three separate measurements ± SD. DEVD represents the nanoprobe, and DEVG represents the control-nanoprobe.

Selectivity studies

To test the detection selectivity of the nanoprobe, 10 μM nanoprobe was incubated with KCl (150 mM), $CaCl_2$ (1 mM), $MgCl_2$ (2.5 mM), glucose (10 mM), glutamine (1 mM), serine (1 mM), arginine (1 mM), vitamin B (1 mM), bovine serum albumin (BSA, 100 nM), GSH (10 mM) trypsin (2 ng mL^{-1}), caspase-8 (another member of caspases; 2 ng mL^{-1}) or caspase-3 (2 ng mL^{-1}) in parallel under the same conditions (37 °C for 24 h). After centrifugation, the supernatant was measured using ICP-MS.

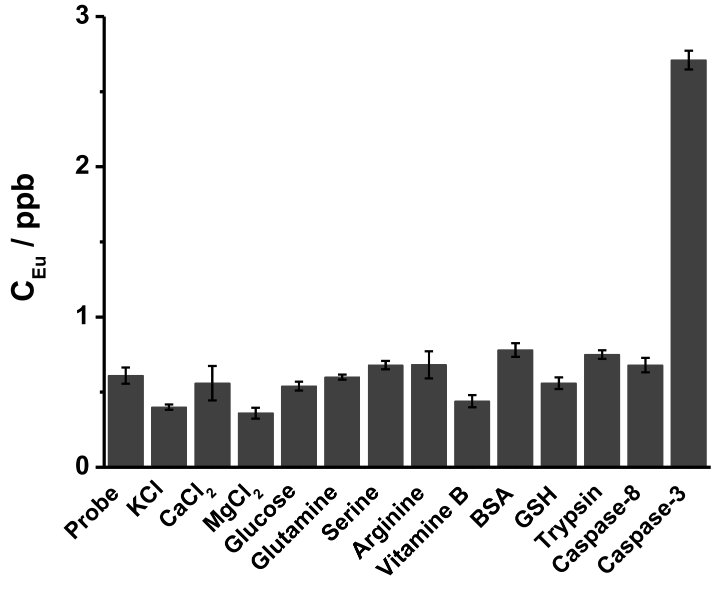

Fig. S9 ICP-MS responses of 10 μM nanoprobe to various substances: KCl (150 mM), $CaCl_2$ (1 mM), $MgCl_2$ (2.5 mM), glucose (10 mM), glutamine (1 mM), serine (1 mM), arginine (1 mM), vitamin B (1 mM), bovine serum

albumin (BSA, 100 nM), GSH (10 mM), trypsin (2 ng mL^{-1}), caspase-8 (another member of caspases; 2 ng mL^{-1}) and caspase-3 (2 ng mL^{-1}). The data represent the mean of three separate measurements ± SD.

Living cell imaging of endogenous active caspase-3

HeLa cells used in this study were cultured in DMEM containing 10% (v/v) fetal bovine serum and 1% (v/v) penicillin−streptomycin at 37 °C in a 5% CO_2 incubator. For fluorescence imaging, the adherent cells grown on glass-bottom culture dishes were incubated with 10 μM nanoprobe or control-nanoprobe in 1 mL of culture media at 37 °C for 12 h. Next, the medium was replaced with fresh medium containing STS of different concentrations or Ac-DEVD-CHO, and the cells were incubated for additional 4 h at 37 °C. Cell imaging was carried out after washing the cells thoroughly with 0.1 M PBS solution (pH 7.4) for three times.

Quantification of endogenous active caspase-3 in HeLa cells

Before quantitative analysis, we first tested the Eu concentration in the supernatant after treated the nanoprobe in lysis conditions in the absence of caspase-3 and centrifuged the sample. The Eu concentration from the nanoprobe with lysis treatment was only 0.72 ppb, which is very close to that of the nanoprobe without lysis treatment (0.61 ppb), demonstrating the nanoprobe is stable in the lysis condition and the non-caspase-3 released Eu is low. The cells were cultured and treated using the same procedures as mentioned above. Next, the cells were washed three times with PBS buffer and trypsinized to remove them from the bottom of the culture dishes for caspase-3 quantification. Densities of the cells in the culture media were measured by cell counting chamber. After centrifugation at 10,000 rpm × 10 min at 4 °C for three times to remove the culture media, the cells were lysated in PBS on the ice with ultrasonication. The lysis was centrifuged at 120,000 rpm × 10 min at 4 °C. After that, the supernatant was subjected to ICP-MS analysis for Eu quantification.

In the data analysis, HeLa cells incubated with the control nanoprobe (with DEVG peptide sequence) and STS were used as the "blank". Since caspase-3 is unable to recognize and cleave DEVG, the Eu signal found in the supernatant of the "blank" sample can be considered as the background signal. The Eu signal obtained using the nanoprobe (with DEVD peptide sequence) was subtracted by the background Eu signal obtained using the control nanoprobe (DEVG), and then substituted in the linear equation ([Eu] (ppb) = 0.98 × [caspase-3] (ng mL^{-1}) – 0.31) from Fig. 2 to calculate the concentration of exogenous caspse-3.

References

S1. X. Wang, Y. Q. Xia, Y. Y. Liu, W. X. Qi, Q. Q. Sun, Q. Zhao and B. Tang, *Chem. Eur. J.*, 2012, **18**, 7189-7195.

S2. F. B. Wu, S. Q. Han, C. Zhang and Y. F. He, *Anal. Chem*., 2002, **74**, 5882-5889.

S3. D. Feng, W. Shi, X. Li and H. Ma, *Chem. Commun*., 2010, **46**, 9203-9205.
